# Dissolved organic matter uptake by *Trichodesmium* in the Southwest Pacific

**DOI:** 10.1038/srep41315

**Published:** 2017-01-24

**Authors:** Mar Benavides, Hugo Berthelot, Solange Duhamel, Patrick Raimbault, Sophie Bonnet

**Affiliations:** 1 Aix Marseille Université, CNRS/INSU, Université de Toulon, IRD, Mediterranean Institute of Oceanography (MIO) UM 110, 98848, Noumea, New Caledonia; 2 Aix Marseille Université, CNRS/INSU, Université de Toulon, IRD, Mediterranean Institute of Oceanography (MIO) UM 110, 13288, Marseille, France; 3 Lamont-Doherty Earth Observatory, Division of Biology and Paleo Environment, Columbia University, PO Box 1000, 61 Route 9W, Palisades, New York 10964, USA; 4 Marine Biological Section, Department of Biology, University of Copenhagen, 3000 Helsingør, Denmark

## Abstract

The globally distributed diazotroph *Trichodesmium* contributes importantly to nitrogen inputs in the oligotrophic oceans. Sites of dissolved organic matter (DOM) accumulation could promote the mixotrophic nutrition of *Trichodesmium* when inorganic nutrients are scarce. Nano-scale secondary ion mass spectrometry (nanoSIMS) analyses of individual trichomes sampled in the South Pacific Ocean, showed significant ^13^C-enrichments after incubation with either ^13^C-labeled carbohydrates or amino acids. These results suggest that DOM could be directly taken up by *Trichodesmium* or primarily consumed by heterotrophic epibiont bacteria that ultimately transfer reduced DOM compounds to their host trichomes. Although the addition of carbohydrates or amino acids did not significantly affect bulk N_2_ fixation rates, N_2_ fixation was enhanced by amino acids in individual colonies of *Trichodesmium.* We discuss the ecological advantages of DOM use by *Trichodesmium* as an alternative to autotrophic nutrition in oligotrophic open ocean waters.

Nitrogen is recognized as the proximate limiting nutrient for primary production in the oceans^1^. The oceanic nitrogen reservoir is controlled by a balance between fixed nitrogen gains (via dinitrogen -N_2_- fixation) and losses (denitrification)^2^. While global nitrogen budget estimations determine that denitrification exceeds N_2_ fixation considerably^3^, recent improvements in the ^15^N_2_ isotope tracer method used to measure biological N_2_ fixation have evidenced that formerly published rates could be underestimated by a factor of ~2 to 6^4^,^5^,^6^,^7^,^8^, and thus could be high enough to balance denitrification on a global basis. However, the variability among N_2_ fixation rates obtained when using the two different methods (adding ^15^N_2_ as a bubble or pre-dissolved in seawater)^4^,^9^ can be high^7^ and at times not significant^10^,^11^,^12^. A mechanistic understanding of which factors determine the degree of discrepancy between the two ^15^N_2_ methods is currently lacking. Moreover, marine pelagic N_2_ fixation had been long attributed to the tropical and subtropical latitudinal bands of the ocean, e.g.^13^, while other ecological niches such as high latitude waters, oxygen minimum zones and the vast dark realm of the ocean (below the euphotic zone) are now recognized as active N_2_ fixation sites^14^,^15^,^16^. It is likely that the inclusion of these previously unaccounted for active N_2_ fixation sites will be more important in equilibrating denitrification and N_2_ fixation rates than the underestimation of rates due to discrepancies between isotopic tracer methods.

In chronically stratified open ocean regions such as the vast subtropical gyres, primary production depends largely on external fixed nitrogen inputs provided by N_2_ fixation performed by prokaryotes termed ‘diazotrophs’. Diazotrophic cyanobacteria are photosynthetic prokaryotes (with the exception of the photoheterotrophic *Candidatus* Atelocyanobacterium thalassa which cannot photosynthesize)^17^ that thrive in oligotrophic tropical and subtropical waters of the oceans where they provide an important source of fixed nitrogen for other phytoplankton^13^. Despite being classically regarded as photoautotrophs, some unicellular diazotrophic cyanobacteria like *Cyanothece* are able to take up dissolved organic matter (DOM) molecules photoheterotrophically^18^. As well, various filamentous diazotrophic cyanobacteria such as *Anabaena* bear genes for amino acids transport, which may be used to incorporate amino acids from the *in situ* DOM pool, or to assimilate amino acids self-produced during diazotrophic growth^19^.

The filamentous diazotrophic cyanobacterium *Trichodesmium* is ubiquitous in the tropical and subtropical oceans where it is estimated to contribute 60–80% of global N_2_ fixation inputs^20^. *Trichodesmium* is limited by iron and/or phosphate availability^21^ which are often scarce in oligotrophic subtropical gyres. The concomitant accumulation of DOM in these oligotrophic gyres^22^ where *Trichodesmium* thrives, suggests it could benefit from organic compounds. While most of the marine DOM is composed of refractory molecules that persist in seawater for millennia, labile DOM (degraded within hours or days) accumulates preferentially at the surface ocean as a result of photosynthesis products^23^. *Trichodesmium* has been shown to assimilate organic phosphorus compounds such as phosphomonoesters and phosphonates when phosphate is scarce^24^,^25^ in an equally efficient manner to phosphate consumption^26^, although a minimum availability of inorganic nutrients may be needed before *Trichodesmium* can cleave the carbon-phosphorus bond of phosphonates^27^. On the other hand, while the uptake of carbon or nitrogen-rich DOM compounds has been studied in cultures of *Trichodesmium* (e.g. refs 28 and 29), such activity has not been revisited for almost two decades. The extent to which mixotrophic nutrition facilitates the growth and/or N_2_ fixation in *Trichodesmium* remains poorly known, particularly for natural colonies. Here we quantify the uptake of carbohydrates and amino acids and their effect on N_2_ fixation by natural *Trichodesmium* colonies using nano-scale secondary ion mass spectrometry (nanoSIMS).

## Results

Station LDA presented relatively oligotrophic conditions at the surface with inorganic nutrient concentrations below the detection limit (0.02 μM for both nitrate -NO_3_^−^- and phosphate -PO_4_^3−^-; Table S1), but high dissolved organic carbon (DOC; 95.34 ± 2.81 μM) and relatively high chlorophyll *a* concentrations (0.36 ± 0.05 μg L^−1^; Table S1) when compared to typical open ocean regional values^30^. Station LDB was sampled in an elevated chlorophyll *a* patch (0.83 ± 0.07 μg L^−1^) and exhibited lower DOC concentrations (70.65 ± 0.09 μM). Bacterial abundance was > 3-fold higher at LDB than at LDA (Table S1).

**Figure 1:**
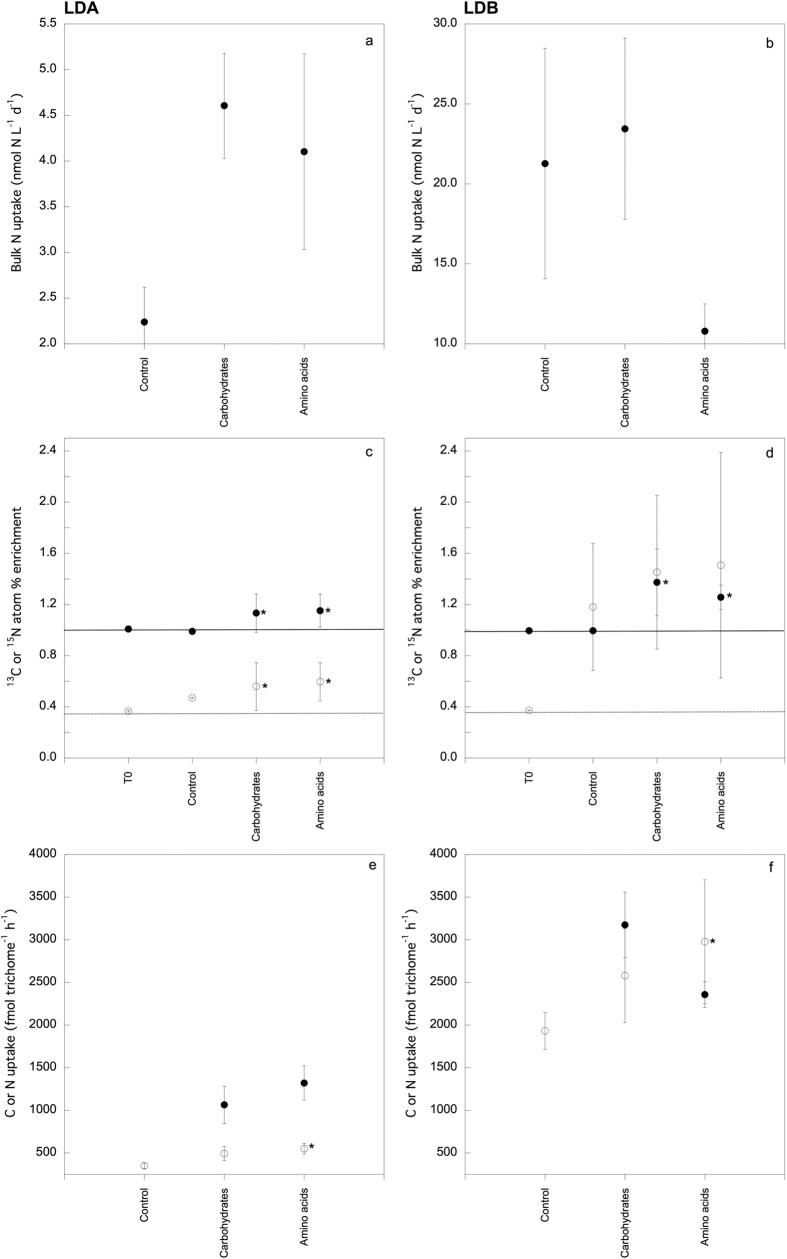
Bulk seawater N uptake rates at (**a**) station LDA, and (**b**) station LDB (note the different scale ranges on the y-axis). *Trichodesmium* (**c,d**) ^13^C -filled circles- and ^15^N -open circles- atom % enrichment values, and (**e,f**) ^13^C and ^15^N uptake rates at stations LDA and LDB, respectively. Error bars for C or N uptake rates (**a,b,e,f**) represent the standard error, while error bars for atom % graphs (**c,d**) represent the standard deviation of the mean. Straight and dotted lines in (**c,d**) indicate the average ^13^C and ^15^N atom % enrichment, respectively, of time zero samples (average natural abundance). Asterisks indicate statistically significant differences (Mann Whitney test, p < 0.05).

Bulk N_2_ fixation rates at LDA were 2.23, 4.61 and 4.10 nmol N L^−1^ d^−1^ for the control, carbohydrate and amino acid treatments, respectively. At station LDB, bulk N_2_ fixation rates were ~9-, 5- and 2-fold higher than at LDA (21.28, 23.44 and 10.79 nmol N L^−1^ d^−1^, respectively; Fig. 1a,b). At LDA, the addition of both carbohydrates and amino acids increased bulk N_2_ fixation but the variability among replicates was high, resulting in non-significant differences (p > 0.05) as observed in previous similar experiments^15^. No significant enhancement of bulk N_2_ fixation rates were observed at station LDB for either treatment.

**Figure 2:**
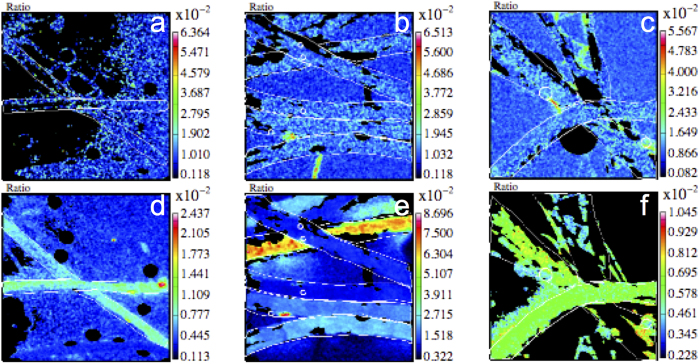
NanoSIMS trichome ^13^C/^12^C ratio images of (**a**) control, (**b**) carbohydrate-amended and (**c**) amino acid-amended samples. The corresponding ^15^N/^14^N ratio images are displayed below (**d–f**).

At both stations LDA and LDB, nanoSIMS analyses of individual trichomes (Table S2) revealed significant ^13^C-enrichments (p < 0.0001) by ~1.7-fold relative to the control upon both carbohydrate and amino acid additions (Fig. 1c,d). These additions also significantly enhanced the ^15^N-enrichment of *Trichodesmium* by ~1.2-fold at station LDA (both p < 0.0001), but not at station LDB, where a high degree of variability was observed between filaments (Fig. 1c,d). When comparing both stations, we observed that per-trichome carbon and nitrogen uptake rates were ~2- and 5-fold higher at LDB than at LDA (Fig. 1e,f). NanoSIMS example images of ^13^C and ^15^N enriched trichomes in control, carbohydrate and amino acid treatments are shown in Fig. 2a–f, respectively. The addition of both carbohydrate or amino acids enhanced per-trichome nitrogen uptake rates at each station, although increases were only statistically significant for amino acid additions (Fig. 1e,f).

## Discussion

We present evidence of carbohydrate and amino acid uptake by natural *Trichodesmium* colonies in conditions usually regarded as optimal for their autotrophic growth (Table S1). The addition of either carbohydrates or amino acids increased per-trichome N_2_ fixation rates compared to the control at both LDA and LDB, but only amino acid additions induced statistically significant per-trichome N_2_ fixation enhancements (Fig. 1e,f). At LDB, the enhancement of per-trichome N_2_ fixation rates with respect to the control upon the addition of either carbohydrates or amino acids was ~5-fold higher than at LDA, suggesting different DOM degradation patterns at LDB. LDB was located inside a massive chlorophyll patch, which had been drifting eastwards for several months (see chlorophyll *a* satellite images time lapse, where station LDB is represented by a pink cross; de Verneil, 2015). The persistence of the patch could be maintained by a regular input of inorganic nutrients via wet deposition, which often enhances primary production due to the high nutrient and trace metal content of volcanic ashes in this highly seismic active area of the South Pacific Ocean^31^. The wet deposition of inorganic nutrients could maintain both photosynthetic and N_2_ fixation activities while promoting a dynamic production and consumption of DOM. The higher DOM uptake by *Trichodesmium* at LDB (Fig. 1d,f) suggests that higher *in situ* DOM availability at LDB compared to LDA, promoted the heterotrophic nutrition of *Trichodesmium* over growth on inorganic nutrients. DOC standing stocks were lower at LDB than at LDA likely due to a tight coupling between its production and consumption, as suggested by the higher bacterial abundance (Table S1) and production at LDB than at LDA (F. Van Wambeke, personal communication). Alternatively, differences in *Trichodesmium* DOM uptake between stations LDA and LDB could be influenced by different DOM uptake genetic ability in different *Trichodesmium* strains^32^.

Given the high energetic cost of CO_2_ and N_2_ fixation in cyanobacteria^33^,^34^, the alternative nutrition on DOM is thought to alleviate energy shortages. For example, unicellular cyanobacteria use glycerol as an alternative to CO_2_^18^, but the use of organic carbon substrates such as carbohydrates by *Trichodesmium* has seldom been observed and at low rates^35^. Although the external input of combined nitrogen is thought to preclude N_2_ fixation in *Trichodesmium*^29^,^36^,^37^, our results show a significant enhancement of per-trichome N_2_ fixation rates upon the addition of amino acids, as observed in other sites where heterotrophic diazotrophs predominate, like in mesopelagic waters^15^,^38^,^39^. Amino acids may provide a more readily accessible source of organic carbon than carbohydrates, resulting in a greater enhancement of N_2_ fixation rates.

DOM utilization likely confers nutritional plasticity to *Trichodesmium* in oligotrophic environments, reinforcing the obsoleteness of the categorical division of marine microbes into autotrophs or heterotrophs. Although we did not conduct ^13^C-labeled bicarbonate uptake experiments during this cruise, previous experiments performed in the Southwest Pacific Ocean and in cultures of *Trichodesmium* IMS101 have shown per trichome bicarbonate uptake rates of ~2–3 × 10^6^ fmol C trichome^−1^ h^−1^ 40,41, which are in the same order of magnitude as the per trichome carbohydrate uptake rates measured here (~1–9 × 10^6^ fmol C trichome^−1^ h^−1^). This suggests that under certain environmental conditions, *Trichodesmium* may be able to exploit carbon comparably from inorganic and organic carbon sources.

Our results cannot however confirm whether DOM molecules were directly taken up by *Trichodesmium*, or if they were primarily reduced by epibiont bacteria and then transferred to the trichomes. For example, heterotrophic bacterial epibionts are known to facilitate dissolved organic phosphorus acquisition in *Trichodesmium* colonies^42^. Thus, the degree and/or functional diversity of epibiont bacteria colonization among sampling stations could have also influenced DOM uptake rates in our *Trichodesmium* samples^43^. We observed bacteria appearing to be attached to trichomes in our samples (Fig. S1), and thus cannot rule out this possibility. Different incubation time span experiments are needed to discern whether DOM passes through bacteria before being taken up by *Trichodesmium*, or if *Trichodesmium* assimilates DOM directly. However, such short-term experiments would require a high isotopic enrichment of the source DOM pool, which would likely bias the measured uptake rates.

We present evidence of carbohydrate and amino acid uptake by natural *Trichodesmium* colonies. Climate change scenarios predict inorganic nutrient limitation and increased DOM retention within the photic zone^44^, which will likely promote mixotrophy in *Trichodesmium*. Further studies on *Trichodesmium* organic versus inorganic nutrient acquisition are thus needed to predict how this important diazotroph will respond to climate alterations.

## Methods

We sampled seawater at two stations in the Southwest Pacific (LDA: 19.21°S-164.68°E, LDB: 18.24°S-170.80°W, on 26 February and 15 March 2015, respectively) at depths receiving 50% of surface photosynthetically active radiation (corresponding to 7 and 9 m depth, respectively). The samples were incubated under *in situ* simulated conditions for 36 h with equimolar quantities of ^13^C-labeled carbohydrates (sodium pyruvate, sodium acetate and glucose) or amino acids (alanine, leucine and glutamic acid; Sigma-Aldrich, Munich, Germany), added at concentrations of 4 μM C (final concentration for the mix of all three carbohydrates or all three amino acids^38^). While the real marine DOM pool is molecularly highly complex and mostly refractory, these commercially available compounds were chosen as representative of carbohydrate and small organic acids typically found in marine labile DOM^15^,^38^,^45^. Seawater was distributed into sixteen 4.3 L transparent polycarbonate bottles (Nalgene, Rochester, NY, USA). Four bottles were filtered immediately upon collection (T0), four were amended with the carbohydrate mix, and another four with the amino acids mix. The last four bottles were used as a control without amendments. All bottles were labeled with 6 mL 98.9 atom% ^15^N_2_ gas (Cambridge Isotope Laboratories, Tewksbury, MA, USA) to assay N_2_ fixation simultaneously. Of each quadruplicate set, three bottles were used to estimate bulk N_2_ fixation rates (expressed as ‘N uptake’) and one bottle was used for nanoSIMS analyses (see Supplementary Information). Mann-Whitney statistical tests were used to test the significance of our results.

## Additional Information

**How to cite this article**: Benavides, M. *et al*. Dissolved organic matter uptake by *Trichodesmium* in the Southwest Pacific. *Sci. Rep.* **7**, 41315; doi: 10.1038/srep41315 (2017).

**Publisher's note:** Springer Nature remains neutral with regard to jurisdictional claims in published maps and institutional affiliations.

## Supplementary Material

Supplementary Information
