# Supplementary material for: Dissolved organic matter uptake by Trichodesmium in the Southwest Pacific
Source: Sci Rep. 2017 Jan 24;7:41315. doi: 10.1038/srep41315 (PMC5259775; doi:10.1038/srep41315)
Supplement: Supplementary Information [file srep41315-s1.pdf]

Dissolved organic matter uptake by *Trichodesmium* in the Southwest Pacific

[1,*]*Mar Benavides,* [2]*Hugo Berthelot,* [3]*Solange Duhamel,* [2]*Patrick Raimbault,* [1]*Sophie Bonnet*

[1]Aix Marseille Université, CNRS/INSU, Université de Toulon, IRD, Mediterranean Institute of Oceanography (MIO) UM 110, 98848, Noumea, New Caledonia

[2]Aix Marseille Université, CNRS/INSU, Université de Toulon, IRD, Mediterranean Institute of Oceanography (MIO) UM 110, 13288, Marseille, France

[3]Lamont-Doherty Earth Observatory, Division of Biology and Paleo Environment, Columbia University, PO Box 1000, 61 Route 9W, Palisades, New York 10964, USA

[*]Corresponding author: Mar Benavides mar.benavides@ird.fr

Supplementary Methods

Hydrography, chlorophyll a*, inorganic and organic nutrients*

Samples were collected during the OUTPACE cruise onboard the R/V *L'Atalante* using a General Oceanics rosette frame fitted with 24 - 12 L Niskin bottles and conductivity, temperature and depth sensors.

Samples for the determination of inorganic nutrients (nitrate (NO_3^-), nitrite (NO_2^-) and phosphate (PO_4^{3-})) were collected in 20 mL acid-washed polyethylene flasks, poisoned with 1% $HgCl_2$, and immediately analyzed onboard using a AA3 Bran+Luebbe autoanalyzer [1]. The detection limit for both NO_3^- and PO_4^{3-} was 0.02 µM. Samples for dissolved organic C (DOC) were collected in combusted glass bottles and immediately filtered through precombusted (4 h, 450ºC) 25 mm glass-fiber filters (GF/F, Whatman, Maidstone, UK) using

a custom-made all-glass filtration system. Filtered seawater was then transferred to precombusted glass ampoules and acidified to pH 2 with 50 µL of 50% phosphoric acid. The samples were analyzed by high temperature catalytic oxidation on a TOC-V analyzer (Shimadzu Scientific Instruments, Columbia, MD, USA) according to Sohrin et al. [2]. Samples for total N (TN) and total P (TP) concentrations determination were collected in 40 mL glass bottles and stored at -20ºC until analysis. TN and TP concentrations were determined according to the wet oxidation procedure as described in Pujo-Pay and Raimbault [3]. DON and DOP concentrations were then obtained by subtracting nitrate plus nitrite concentrations from TN (i.e. TN-NO_3^--NO_2^- = DON), and DOP by subtracting phosphate concentrations from TP (i.e. TP-PO_4^{3-} = DOP). Chlorophyll (Chl *a*) concentrations were determined from 500 mL samples filtered through GF/F filters. Chl a was extracted in methanol and measured by fluorometry [4].

$^{15}N_2$ labeling and isotope ratio mass spectrometry analyses

After ^{13}C additions, control, carbohydrate and amino acid treatment bottles were filled to overflow and closed without bubbles using screwcaps fitted with teflon-lined rubber septa. Six mL of high purity $^{15}N_2$ gas (Cambridge Isotope Laboratories, Tewksbury, MA, USA) were injected through the septum using a gas-tight syringe as detailed in Montoya et al. [5]. The bottles were inverted 20 times to facilitate the dissolution of $^{15}N_2$ gas. All treatment bottles (control, carbohydrate amino acid treatments) were incubated in on-deck incubators at the appropriate irradiance with continuously flowing surface seawater for 36 h. The $^{15}N_2$ tracer method is susceptible of both underestimations and overestimations. The slow dissolution of the $^{15}N_2$ bubble into the seawater results in a lower than theoretically calculated initial dissolved N_2 concentration in the sample, which in turn leads to an underestimation of N_2 fixation rates [6]. This problem can be solved by pre-dissolving $^{15}N_2$ in the sample seawater

and, most importantly, measuring the real (and not theoretically calculated) value of dissolved N_2 in the sample [6-8]. Because the preparation of $^{15}N_2$-enriched filtered seawater to be used as a tracer requires considerable sample manipulation and often leads to trace metal [9] and DOC contamination (N. Wannicke, personal communication), we chose instead to measure the $^{15}N/^{14}N$ ratio of dissolved N_2 in our samples before and after the incubation period by membrane inlet mass spectrometry (MIMS [10]), and apply the average value of the hundreds of measurements made along the cruise (6.15 ± 0.79 atom %, data not shown) to calculate N_2 fixation rates. On the other hand, N_2 fixation rates may be overestimated if the stock $^{15}N_2$ gas used is contaminated with inorganic nitrogen forms such as NO_3^- or ammonium [11]. The $^{15}N_2$ gas used in this study was pre-checked in the R. Dabundo's lab (University of Connecticut, USA) before the cruise and its contamination with inorganic nitrogen forms other than N_2 was found to be minimal, resulting in a negligible overestimation of N_2 fixation rates (<1%, see [12]).

Of each quadruplicate set of incubation bottles, one bottle was kept for nanoSIMS analyses (see below). The three remaining bottles were filtered onto pre-combusted (450ºC, 4 h) Whatman GF/F filters (Whatman, St. Louis, MO, USA) and stored at -20ºC until further analysis. Prior to analysis, the filters were dried at 60ºC for 24 h and packed in tin foil cups. The samples were analyzed by continuous-flow isotope ratio mass spectrometry using an Integra2 Analyzer (Integra CN, SerCon Ltd, Cheshire, UK), which was calibrated every ten samples using reference material (International Atomic Energy Agency, IAEA-N1). N_2 fixation rates were calculated as specified in Montoya *et al.* [5].

nanoSIMS analyses

At the end of the incubation period, the content of one bottle per treatment was filtered through 10 μm pore size 25 mm diameter polycarbonate filters (Millipore, Darmstadt,

Germany) using a peristaltic pump. The filters were fixed with 4% paraformaldehyde (final concentration) prepared in 0.2 µm filtered seawater for 30 min and stored in petri dishes at -80ºC until analysis.

Circles of 1 cm diameter were excised from the 10 µm polycarbonate filters and placed onto nanoSIMS sample holders. The analyses were performed using a N50 nanoSIMS (Cameca, Gennevilliers, France) at the French National Ion MicroProbe Facility according to previously described methods [13-15]. A 1.3 to 3 pA 16 keV Cesium (Cs^+) primary beam focused onto ~100 nm spot diameter was scanned on a 256 × 256 pixel raster with a raster area of 40 × 40 µm, and a counting time of 1000 µs per pixel. Samples were pre-sputtered with Cs^+ for 5 - 6 min to remove surface contaminants and increase conductivity. Negative secondary ions $^{12}C^-$, $^{13}C^-$, $^{12}C^{14}N^-$, $^{12}C^{15}N^-$ and $^{31}P^-$ were detected with electron multiplier detectors, and secondary electrons were simultaneously imaged. Twenty serial quantitative secondary ion mass planes were generated and accumulated to the final image. Mass resolving power was ~8000 in order to resolve isobaric interferences. Data were processed using the LIMAGE software (L. Nittler, Carnegie Institution of Washington). All scans were corrected for any drift of the beam during acquisition. Isotope ratio images were generated by dividing the $^{13}C^-$ ion count by the $^{12}C^-$ ion count, and the $^{12}C^{15}N^-$ ion count by the $^{12}C^{14}N^-$ ion count. Individual *Trichodesmium* filaments (trichomes) were easily identified in nanoSIMS secondary electron $^{12}C^-$, $^{12}C^{14}N^-$ images. These images were used to define regions of interest (ROI). For each ROI, the $^{13}C/^{12}C$ and $^{15}N/^{14}N$ ratios were calculated.

Biomass calculations, ^{13}C and ^{15}N assimilation rates per ROI

We considered a biomass of 2.498 nmol C trichome^{-1} as used in Luo *et al.* [16]. Considering a C:N ratio of 6:1 [17], the N content of *Trichodesmium* was 0.416 nmol N trichome^{-1}. *Trichodesmium* C and N assimilation rates were calculated for each trichome

analyzed with the nanoSIMS (i.e. each ROI) as $C\ assimilation = (^{13}C_{XS} \times C_{con})/^{13}C_{sr}$ where $^{13}C_{XS}$ is the ^{13}C atom % excess with respect to T0 values, C_{con} is the C content of each ROI determined as explained above, and $^{13}C_{sr}$ is the ^{13}C atom % enrichment of the source pool (^{13}C-labeled carbohydrate or amino acid mixes). An equivalent expression was used to calculate ^{15}N assimilation with $^{15}N_2$ as the source pool. Assimilation rates were expressed as fmol C or N per trichome and hour.

Scanning electronic microscopy (SEM) sample preparation

Pieces of polycarbonate filters were carefully washed three times with sterile phosphate buffer (pH 7.3) and treated with a staining and fixing protocol adapted from Priester *et al.* [18]. Briefly, the filters were prefixed for 30 min with 0.075% ruthenium red (Sigma-Aldrich, Saint Louis, MI, USA), 50 mM TLC-grade L-Lysine (Sigma) and 2.5% glutaraldehyde in phosphate buffer. Subsequently, the filters were fixed for 2 h with 0.075% ruthenium red and 2.5% glutaraldehyde in phosphate buffer, and then washed three times (10 min each) with phosphate buffer. The filter pieces were transferred to clean Petri dishes, covered to prevent drying and refrigerated (4°C) until observation.

References

1. Aminot, A. & Kérouel, R. *Dosage automatique des nutriments dans les eaux marines: méthodes en flux continu*. (Editions Quae, 2007).

2. Sohrin, R. & Sempéré, R. Seasonal variation in total organic carbon in the northeast Atlantic in 2000–2001. *J Geophys Res* **110,** C10S90 (2005).

3. Pujo-Pay, M. & Raimbault, P. Improvement of the wet-oxidation procedure for simultaneous determination of particulate organic nitrogen and phosphorus collected on filters. *Mar Ecol Prog Ser* **105,** 203–207 (1994).

4. Herbland, A., Le Bouteiller, A. & Raimbault, P. Size structure of phytoplankton biomass in the equatorial Atlantic Ocean. *Deep Sea Research Part A. Oceanographic Research Papers* **32,** 819–836 (1985).

5. Montoya, J. P., Voss, M., Kahler, P. & Capone, D. G. A simple, high-precision, high-sensitivity tracer assay for N_2 fixation. *Appl Environ Microbiol* **62,** 986–993 (1996).

6. Mohr, W., Großkopf, T., Wallace, D. W. R. & LaRoche, J. Methodological Underestimation of Oceanic Nitrogen Fixation Rates. *PLOS ONE* **5,** e12583 (2010).

7. Großkopf, T. *et al.* Doubling of marine dinitrogen-fixation rates based on direct measurements. *Nature* **488,** 361–364 (2012).

8. Wilson, S. T., Böttjer, D., Church, M. J. & Karl, D. M. Comparative Assessment of Nitrogen Fixation Methodologies, Conducted in the Oligotrophic North Pacific Ocean. *Appl Environ Microbiol* **78,** 6516–6523 (2012).

9. Klawonn, I., LaviK, G. & Böning, P. Simple approach for the preparation of $^{15}N_2$-enriched water for nitrogen fixation assessments: Evaluation, application and recommendations. *FMICB* **6,** 769 (2015).

10. Kana, T. M. *et al.* Membrane inlet mass spectrometer for rapid high-precision determination of N_2, O_2, and Ar in environmental water samples. *Anal. Chem.* **66,** 4166–4170 (1994).

11. Dabundo, R. *et al.* The Contamination of Commercial $^{15}N_2$ Gas Stocks with ^{15}N-Labeled Nitrate and Ammonium and Consequences for Nitrogen Fixation Measurements. *PLOS ONE* **9,** e110335 (2014).

12. Benavides, M. *et al.* Mesopelagic N_2 Fixation Related to Organic Matter Composition in the Solomon and Bismarck Seas (Southwest Pacific). *PLOS ONE* **10,** e0143775 (2015).

13. Musat, N. *et al.* A single-cell view on the ecophysiology of anaerobic phototrophic

bacteria. *Proc. Natl. Acad. Sci. U. S. A.* **105,** 17861–17866 (2008).

14. Foster, R. A. *et al.* Nitrogen fixation and transfer in open ocean diatom–cyanobacterial symbioses. *ISME J* **5,** 1484–1493 (2011).

15. Foster, R. A., Sztejrenszus, S. & Kuypers, M. M. M. Measuring carbon and N2 fixation in field populations of colonial and free-living unicellular cyanobacteria using nanometer-scale secondary ion mass spectrometry 1. *J Phycol* **49,** 502–516 (2013).

16. Luo, Y. W. *et al.* Database of diazotrophs in global ocean: abundance, biomass and nitrogen fixation rates. *Earth Syst. Sci. Data* **4,** 47–73 (2012).

17. Redfield, A. C. *et al.* in *The Sea* 26–77 (Interscience, 1963).

18. Priester, J. H., Horst, A. M. & Van De Werfhorst, L. C. Enhanced visualization of microbial biofilms by staining and environmental scanning electron microscopy. *Journal of Microbiological Methods* **68,** 577–587 (2007).

Supplementary Figures

Fig. S1: Example of SEM image of a *Trichodesmium* filament with epibiotic bacteria.

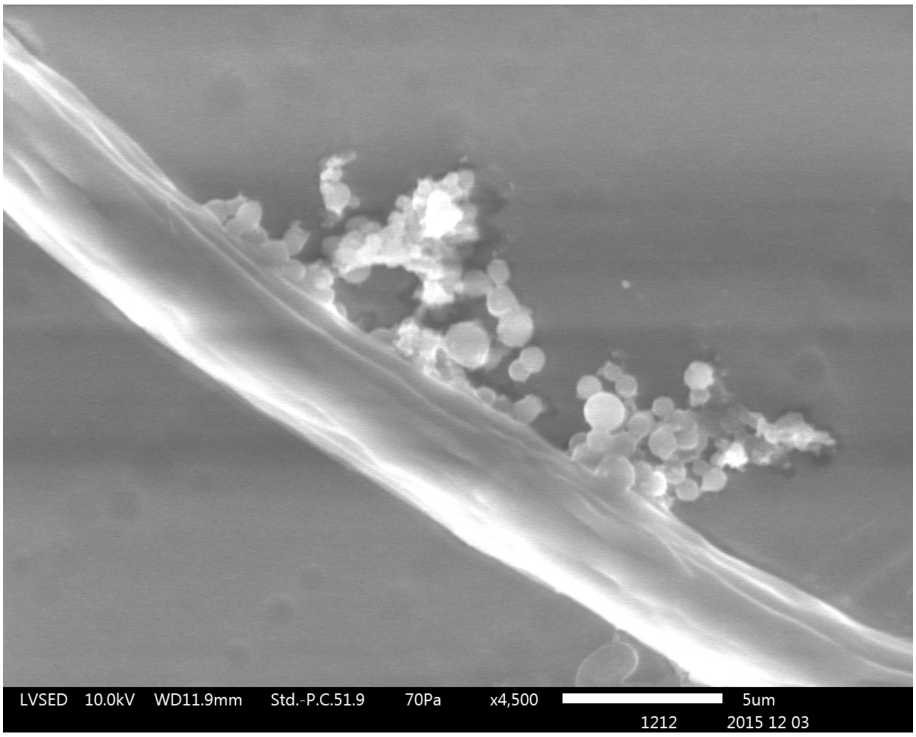

Table S1: Values of temperature, salinity, nitrate (NO_3^-), phosphate (PO_4^{3-}), dissolved organic carbon, nitrogen and phosphorus (DOC, DON and DOP, respectively), chlorophyll *a,* dissolved iron (dFe) and bacterial abundance measured at the start (T0) of experiments. n/d means not analytically detectable.

Station	Temperature (ºC)	Salinity	NO_3^- (µM)	PO_4^{3-} (µM)	DOC (µM)	DON (µM)	DOP (µM)	Chlorophyll *a* (µg L^{-1})	dFe (nM)	Bacteria abundance (cells mL^{-1})
LDA	29.2	34.84	n/d	n/d	95.34±2.81	6.20±0.51	0.18±0.02	0.36±0.05	0.85	3.74 x 10^5
LDB	29.9	35.05	n/d	n/d	70.65±0.09	6.09±0.58	0.18±0.02	0.83±0.07	0.71	12.9 x 10^5

Table S2: Number of trichomes analyzed by nanoSIMS for ^{13}C and ^{15}N atom % enrichment.

Station	Treatment	Number of trichomes
LDA	T0	15
	Control	33
	Carbohydrates	33
	Amino acids	32
LDB	T0	22
	Control	30
	Carbohydrates	33
	Amino acids	33
